# An Electrochemical Study of Two Self-Dopable Water-Soluble Aniline Derivatives: Electrochemical Deposition of Copolymers

**DOI:** 10.1155/2012/737013

**Published:** 2012-04-02

**Authors:** Loredana Vacareanu, Ana-Maria Catargiu, Mircea Grigoras

**Affiliations:** Electroactive Polymers Department, “P. Poni” Institute of Macromolecular Chemistry, 41A Gr. Ghica Voda Alley, 700487 Iasi, Romania

## Abstract

An electrochemical study of two water-soluble aniline derivatives, *N*-(3-sulfopropyl) aniline (AnPS) and *N*-(3-sulfopropyl) *p*-aminodiphenylamine (DAnPS), in aqueous acidic electrolytic solutions containing different kinds of doping anions (Cl ^−^, SO_4_^2−^, and ClO_4_^−^) was carried out. At sufficiently high anodic potential, the sulfonated aniline derivatives undergo oxidation processes yielding cation-radical and dimer intermediates, but no polymer deposition was observed on the working electrode surface. Experimental results showed that both aniline derivatives are electroactive compounds exhibiting redox behaviour in the range of potential of −0.2 V–1.6 V. Due to the self-doping effect induced by sulfonic groups, AnPS and DAnPS compounds have good electroactivity even in neat water solution. By adding a small amount of aniline into electrolytic system, thin layers of copolymers were deposited on the working electrode surface. The copolymer layers formed on the electrodes show a highly orientational and positional order, confirmed by AFM and XRD spectroscopic techniques. During the anodic oxidation processes some distinct colour changes were observed.

## 1. Introduction

Polyaniline (PANI) is one of the most promising materials from a technological standpoint because it hacs a high conductivity, can be easily synthesized, has well-behaved electrochemistry (the colour changes depending on the redox state), and is stable under ambient conditions. However, the low solubility in common organic solvents and the strong dependence of its electrical conductivity on the pH of the electrolytic medium (which falls almost to zero at pH higher than 3) [1] are inherent problems that should be overcome [2]. In this context, great efforts have been made towards the synthesis of modified structure of the PAni, either from (electro)polymerization of suitable substituted monomers [3–8] or from the interaction of functional groups after synthesis of the polymers [9].

The (electro)oxidation mechanism and the kinetics of the poly(*N*-alkylaniline) synthesis in aqueous and nonaqueous solutions have been the subject of numerous investigations devoted to identify the steps of the electropolymerization process [10–14]. An EC mechanism for electrooxidation of *N-*substituted anilines based by *in situ* UV-Vis spectroelectroscopy had been proposed [5–8], and based on this mechanism it had been reported that the formation of primary radical cation is the rate-limiting step in electrochemical oxidation of aniline. The radical-radical coupling, proton release, and further electrochemical oxidation steps lead to the oligomers generation. The high solubility of monomers and polymers based on *N-*alkylaniline substituted with acidic groups in organic medium and even in water is due to the chain length of the alkyl group [15] and to the presence of the acidic groups such as carboxyl [16–20], sulfonic [4–8, 21–25] and phosphoric [26]. Furthermore, the acidic groups can react with imine nitrogen to form polarons, which are the charge carriers of doped PAni. The self-doped effect differs from external doping effect of protonic acid and will provide higher electrochemical activity and electric conductivity than conventional PAni in nonaqueous or neutral and basic system, because self-protonation is independent of external protonation in a broad pH range [25, 26].

In order to obtain sulfonated self-doped polyanilines, possessing different solubility and enhanced properties, the chemical or electrochemical copolymerization reactions are the best ways, by choosing the comonomer ratio and appropriate (electro)polymerization conditions [27]. Fortunately, an addition of a small amount of unsulfonated aniline monomer into a polymerization solution containing sulfonated monomer leads to formation of self-doped copolymers which can be either soluble or insoluble depending on the molar ratio of sulfonated and unsulfonated monomer units in the resulting copolymer backbone.

In this paper we present an electrochemical study of two aniline derivatives, *N-*(3-sulfopropyl) *p*-aminodiphenylamine (DAnPS) and *N-*(3-sulfopropyl) aniline acid (AnPS), in acidic aqueous solutions such as H_2_SO_4_, HCl, and HClO_4_ and even in neat water. Due to the lack of the solubility of the DAnPS, the cyclic voltammograms were recorded in a mixture containing DMF as organic solvent and an acidic aqueous solution. To our knowledge, based on the literature data, the DAnPS's electrochemical properties were not investigated until now.

## 2. Experimental

### 2.1. Materials

Aniline was double-distilled under reduced pressure just before use and stored in the dark and in a nitrogen atmosphere. *N-*(3-sulfopropyl) aniline (AnPS) and *N-*(3-sulfopropyl) *p*-aminodiphenylamine (DAnPS) were synthesized according to the literature [27, 28], and the synthetic procedure and characterization were presented elsewhere [27]. In all electrochemical studies three electrolyte solutions were employed such as hydrochloric acid (HCl), perchloric acid (HClO_4_), and sulphuric acid (H_2_SO_4_), and these were prepared using bidistilled water. Lithium perchlorate (LiClO_4_) used as electrolyte in cyclic voltammetry studies, was purchased from Aldrich. Dimethylformamide (DMF) and other solvents (all from Aldrich) are commercial products and were used as received or dried by usual methods and distilled under reduced pressure.

### 2.2. Instruments

Electrochemical studies of aniline derivatives were carried out using a bioanalytical systems, Potentiostat-Galvanostat (BAS 100 B/W). The experiments were performed in a one-compartment cell using a standard three-electrode cell arrangement, including a working electrode, an auxiliary electrode (platinum wire), and a reference electrode (consisted of a silver wire coated with AgCl). A disk-shape Pt electrode (1.6 mm diameter), Pt rectangular plate (1.0 × 0.5 cm^2^ area) and a square transparent glass-ITO electrode (2.5 × 2.5 cm^2^ area), were used as working electrodes. Before each experiment, the Pt working electrodes were cleaned by polishing successively with 0.3 and 0.05 *μ*m Al_2_O_3_ slurry on emery paper to a mirror finish and then washed with methanol and bidistilled water. The working electrode potential was always measured against an Ag/AgCl reference electrode. The reference electrode (Ag/AgCl) was calibrated at the beginning of the experiments by running the CV of ferrocene as the internal standard in an identical cell without any monomer in the system (*E*_1/2_ = 0.425 V *versus* the Ag/AgCl). Before each experiment the electrolyte solutions were degassed by bubbling argon for 3 minutes. All electrochemical experiments were carried out in stationary solutions and at room temperature (about 25°C).

XRD measurements were performed with a Bruker AD8 ADVANCE diffractometer. The X-ray beam was CuK*α*_1_ (1.5406 Å) radiation operating from a sealed tube operated at 40.0 kV and 30 mA. Data from 3° to 50° (2*θ*) were obtained using Bragg-Brentano geometry at a scan rate 1.0 deg/min.

The morphology of the films was investigated by atomic force microscopy (AFM) with a MultiView 4000 Nanonics System, working in noncontact mode using probes with the resonance frequency of 38–40 kHz.

## 3. Results and Discussions

### 3.1. Electrochemical Behaviour of AnPS and DAnPS

**Figure 1:**
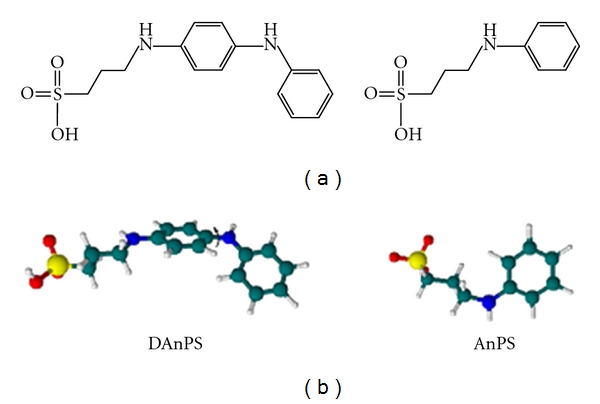
(a) The chemical structures and (b) the optimized structural geometry of the aniline derivatives, DAnPS and AnPS.

The detailed synthesis procedure and structural characterization of the AnPS was reported in the literature [27]. Unlike aniline, AnPS has the advantage water soluble and maintaining its redox activity even at neutral pH. A mechanism for electrooxidation of AnPS, based on *in situ* UV-Vis investigation was already proposed in the literature [25, 28, 29]. The second monomer, DAnPS, was synthesized by ring opening reaction of 1,3-propanesultone and addition to *N-*phenyl-1,4-phenylendiamine in acetonitrile, at 100°C for 12 h. Although there are some reports that describe the spectroelectrochemical behaviour of AnPS [30], the electrochemical behaviour of the *N-*alkyl-sulfonated dimer, DAnPS, was not studied until now, and the redox behaviour is reported here, for the first time. The chemical structures of the aniline derivatives employed in this electrochemical study are depicted in Figure 1.

The DAnPS structure can be viewed as a dimer of aniline with an AnPS unit. Although the presence of the sulfopropyl substituent attached to the nitrogen atom increases the water solubility of AnPS, the DAnPS is insoluble in water due to the hydrophobic effect of the second phenyl substituent. The solubility of DAnPS sample follows the following order: DMSO > DMF *⋙* acidic aqueous solutions. Thus, the electrochemical studies of the DAnPS were performed in a mixture consisting of DMF and aqueous acidic solutions.

**Figure 2:**
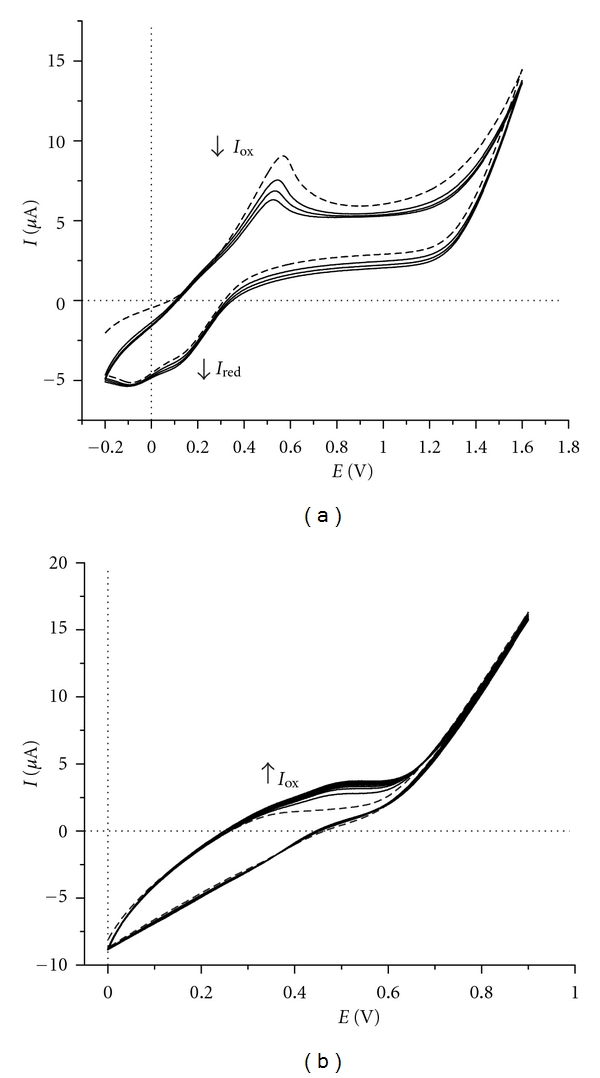
Multicyclic voltammograms of (a) 1.0 mM DAnPS (water dispersion) and (b) 1.0 mM AnPS on Pt electrode (disk-shaped, 1.6 mm diameter) recorded in bidistillated water; the dotted line represents the first cycle. Scan rate: 50 mV s^−1^.

The multicyclic voltammograms of DAnPS and AnPS in bidistillated water (free of any supporting electrolyte) on the disk-shaped Pt electrode were recorded by sweeping the working electrode potential between −0.2 V (0.0 V) and 1.6 V (1.2 V), respectively, at a constant rate of 50 mV s^−1^ (Figure 2). DAnPS has a poor solubility in bidistillated water and forms blue-white water dispersion. By submission of this dispersion to cyclic voltammetry measurements, the current intensity-potential curve recorded at the Pt electrode exhibits one anodic peak and two cathodic peaks corresponding to the oxidation and reduction processes (Figure 2(a)).

By increasing the number of the scans, the anodic current peak intensity decreases. Thus, the first forward scan shows that the onset of oxidation process of DAnPS occurs at 0.118 V, and the peak is centred at 0.569 V (*versus* Ag/AgCl). Some cation-radical species generated during the oxidation process are reduced on the reverse scanning, and two broad cathodic peaks at 0.145 V and −0.091 V are observed.

Unlike DAnPS, the water-soluble AnPS, exhibits only one broad anodic peak on the subsequent cycles (Figure 2(b)). At the end of the 20th cycle, the AnPS-water solution became green and no deposition product was observed on the electrode surface. Soluble ECE reactions products are formed, but they diffuse into the bulk solution.

**Figure 3:**
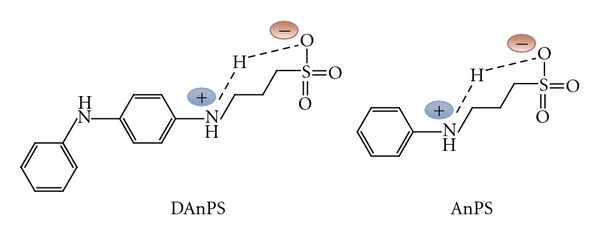
The zwitterionic form of the AnPS and DAnPS monomers in aqueous solution.

Using a solution containing 1.0 mM DAnPS in organic solvent (DMF or DMSO), free of any electrolyte support, the monomer is free of electrochemical activity. This behaviour can be explained by the fact that in water some sulfonated groups (–SO_3_H) exist in a dissociated form giving to monomers zwitterionic structures, while in the organic solvent the sulfonated groups are nondissociated (Figure 3). Thus, in water DAnPS and AnPS derivatives contain in their ionisable structure negatively charged functional group, which acts as an inner dopant anion reducing the need for external dopants. In acidic solutions, the amine and quinonimine groups are charged, and thus the negative charge of the sulfonated group is compensated [28].

**Figure 4:**
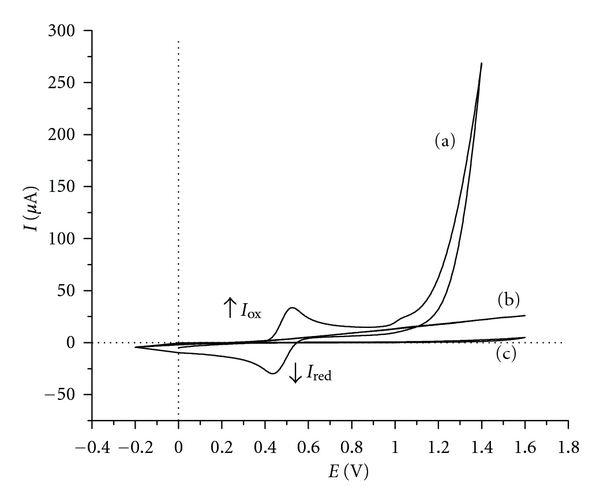
(a) The CV of 1.0 mM DAnPS at Pt electrode (disk shape, 1.6 mm diameter) recorded in DMF and LiClO_4_ as supporting electrolyte; (b) the CV of 1.0 mM DAnPS in DMF-free electrolyte support (LiClO_4_); (c) the CV of the DMF-LiClO_4_ monomer-free solution; scan rate: 50 mV s^−1^.

By using LiClO_4 _as supporting electrolyte, DAnPS exhibits reversible cyclic voltammogram curves (Figure 4). It can be observed that a reversible redox couple appears with an oxidation peak located at 0.525 V and the reduction peak located at 0.435 V (*versus* Ag/AgCl). The midpoint potential (*E* ^0′^) is the average value of anodic and cathodic potential peaks and in this case is 0.480 V.

The second anodic peak is located at 1.027 V and can be assigned to the second oxidation process of DAnPS. No oxidation peak was observed up to a potential of 1.400 V on the anodic scan.

**Figure 5:**
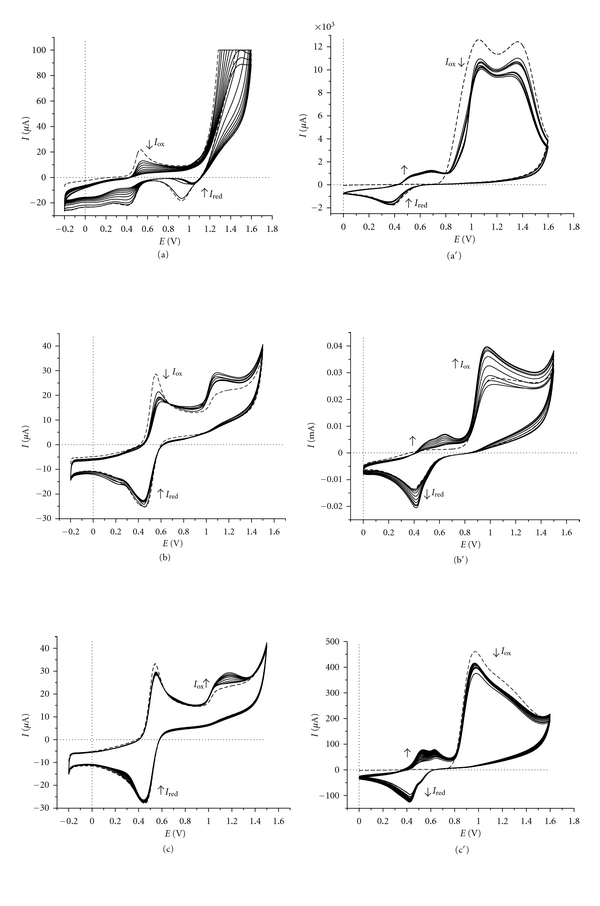
Multicyclic voltammograms (20 cycles) of 1.0 mM DAnPS in (a) DMF-HCl (1 M), (b) DMF-H_2_SO_4_ (1 M) and (c) DMF-HClO_4_ (1 M), and 1.0 mM AnPS at Pt electrode (disk shape,1.6 mm diameter) in (a′) HCl (1 M), (b′) H_2_SO_4_ (1 M) and (c′) HClO_4_ (1 M); the dotted line represents the first cycle. Scan rate: 50 mV s^−1^.

In Figure 5 are presented the multicyclic voltammograms of DAnPS and AnPS, using three acidic aqueous solutions as electrolytic medium. Due to the lack of the solubility in aqueous acidic solvents, the electrochemical characterization of DAnPS was carried out in the mixtures of DMF-acidic aqueous solution. Thus, three electrolytic mixtures: (a) DMF-HCl (1 M), (b) DMF-H_2_SO_4_ (1 M), and (c) DMF-HClO_4_ (1 M) were employed. The potential of the working electrode was swept in the range of −0.2 V–1.6 V, with a scan rate of 50 mV s^−1^.

In DMF-HCl (1 M) mixture, the CV of the DAnPS (1.0 mM) exhibits on the first scan two anodic peaks associated to monomer oxidation processes. The second anodic peak cannot be observed in this CV because its current intensity is very high (Figure 5(a)). After the 7th cycle the anodic currents peak intensities decreased, and the peak associated with the second oxidation processes can be observed at 1.474 V. On the reverse scanning, DAnPS exhibits two cathodic peaks located at 0.431 V and 0.937 V being caused by the reduction of the oxidized species. All currents peak intensities decrease with the increasing of the number of scans. All the electrochemical data are summarized in Table 1.

In H_2_SO_4_ and in HClO_4_ aqueous solutions (1 M), DAnPS exhibits two anodic peaks at 0.552 V and 1.075 V, and 0.544 V and 1.064 V, respectively, on the first scan. On the reverse scanning, only one cathodic peak appears at 0.454 V (in H_2_SO_4_ aqueous solution) and 0.455 V (in HClO_4_ aqueous solution). It can be noticed that the first oxidation-reduction couple is reversible, and this is confirmed by the value of the ratio between the first anodic and cathodic peaks currents intensities of 1.16 (Figure 5(b)) and 1.17 (Figure 5(c)).

Taking into account the electrooxidation mechanism of 4-aminodiphenylamine in acetonitrile, it can be stated that the first one-electron transfer peak of DAnPS gives rise to a cation radical that later undergoes irreversible follow-up deprotonation to give a radical which then undergoes another electron transfer involving an ECE mechanism [31, 32]. In both cases, using H_2_SO_4_ and in HClO_4_ aqueous solutions, on subsequent cycling, a decrease in the intensity of the first oxidation peak current can be noticed, while the intensity of the second oxidation peak current increases. Initially, the colour of the DAnPS-acidic aqueous-DMF mixture is blue and at the end of the 20th cycles became dark-blue coloured, and no deposition product was observed on the working electrode surface. A dark blue soluble reaction product was observed, but it diffuses into the bulk solution.

**Scheme 1:**
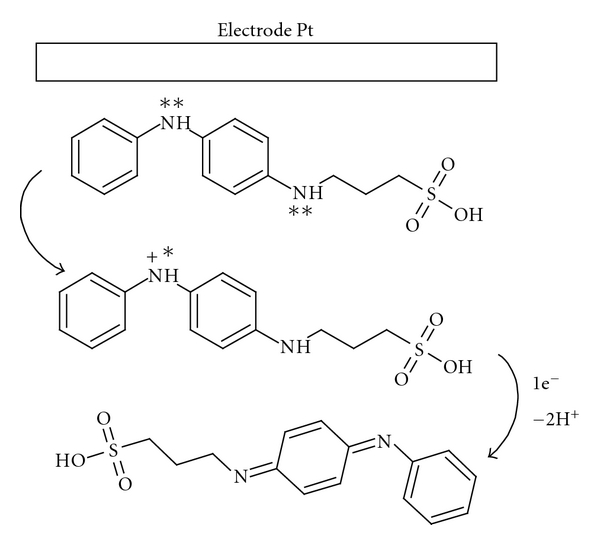
Possible anodic oxidation process of the DAnPS monomer, at the Pt electrode surface.

Scheme 1 presents the proposed mechanism of the oxidation process of the DAnPS monomer, at the Pt electrode.

The negative shift of the first redox couple compared to *N-*phenyl-1,4-phenylendiamine can be explained by the fact that the anodic oxidation of the DAnPS derivative is influenced by the presence of the bulky propyl-sulfonic acid group attached directly to the nitrogen atom. From the electrochemical data reported in the literature, in 1 M HCl and at Pt electrode, anodic oxidation of *N-*phenyl-1, 4-phenylendiamine exhibits one anodic peak centered at around 0.77 V, on the first forward scan, and one small cathodic peak at 0.74 V on the reverse [33]. In other words, it might be possible that only one-electron process occurs at the Pt electrode, in the first stage. The first electron migrates from the free nitrogen atom generating the first anodic peak, due to the different electroactivity of the two nitrogen atoms. The second anodic peak can be generated by the second one-electron oxidation process at the second nitrogen atom from the DAnPS molecule. The propyl-sulfonic group has an electron-withdrawing character which decreases the charge delocalization at the aniline ring, and thus, the electrons from the second nitrogen atom are less available for oxidation. Thus, the second one-electron oxidation process occurs at higher anodic potential than the first one.

In Figure 5 are represented the cyclic voltammograms of the AnPS (1.0 mM) recorded at Pt electrode, in HCl (a′), H_2_SO_4_ (b′), and HClO_4_ (c′) aqueous solution. In HCl (1 M) electrolysis medium, AnPS presents on the first scan two anodic peaks appearing at 1.051 V and 1.363 V, which corresponds to the oxidation processes of the monomer. On the reverse scan, the cathodic peak located at 0.390 V can be assigned to the reduction of the oxidized species at the electrode-solution interface. The low charge recorded for the cathodic peak can be related with the possibility that those molecules that are oxidized at positive potential at the end of the electron-transfer reaction could react through chemical reactions. During the recording of the subsequent cycles in the CVs two new anodic peaks appear at 0.533 V and 0.673 V, and their current intensities increase with increasing of the number of scans. The anodic currents intensity peaks of the first cycle are diminished with increasing the scanning number. The appearance of the new oxidation peaks suggests the formation of new species on the electrode surface, while the monomer is consumed. At the end of the 20th cycle, a bluish-green-coloured product was easily eluted into bulk solution from the electrode. No film was observed on the electrode surface and this can be explained by the fact that the presence of the alkyl chain and the sulfonate groups increase the solubility of the polymer. An explanation for the impossibility of obtaining the electrodeposited polymer film was given by Malinauskas et al. [4–8, 30]. They admit the fact that the oligomer formation from *N-*(3-sulfopropyl) aniline (AnPS) alone should have positively charged nitrogen atom which makes it extremely soluble in water and aqueous electrolytic medium. The deposition of the film was hardly observed even with increasing the monomer concentration.

It is well known that the potential value needed for a polymer, oligomer, or monomer to be oxidized follows this order: *E*_(polymer)_ < *E*_(oligomer)_ < *E*_(benzoquinone)_ < *E*_(dimer)_ < *E*_(monomer)_. Similar consideration can be applied to the substituted monomers reported herein. Since the dimer has a lower potential than the monomer and so the DAnPS is much easier to oxidize than AnPS, it can be stated that those new peaks which appear on the subsequent AnPS's cyclic voltammograms correspond to the AnPS dimer formation on the working electrode.

The cyclic voltammograms of AnPS recorded in H_2_SO_4_ (1 M) and HClO_4_ (1 M) show approximately the same behaviour as in HCl electrolyte medium, except the fact that the second anodic peaks are not very well distinguished, more broad, and overlapped on the first anodic one. This suggests the low stability of the cation-radical intermediate (AnPS^+∗^) formed in the first oxidation. The radical cation intermediate generated at the surface of electrode can react with another cation-radical to form a dimer. The dimer is then oxidized in one step to diimine quinoid form with participation of two electrons, at a lower oxidation potential in comparison with AnPS monomer.

The low stability of the cation-radical intermediate can be related with the presence in the electrolyte medium of SO_4_^2−^ and ClO_4_^−^ anions. Due to the presence of the bulky propyl-sulfonic group attached to the molecule, the large SO_4_^2−^ and ClO_4_^−^ anions could not move easily in order to compensate the negatively charged nitrogen. In case of Cl^−^ anions, the doping process occurred and the intermediate formed during the anodic oxidation is more stable and undergoes further electrooxidation or chemical process. On the reverse scanning, one broad cathodic peak appears at 0.399 V (in H_2_SO_4_ aqueous solution) and 0.522 V (in HClO_4_ aqueous solution) that can be assigned to reduction process of the oxidized species at the Pt surface. In addition, in aqueous solution the first cathodic peak that corresponds to the second anodic one is overlapped on the first cathodic peak or hardly detectable. At the end of the 20th cycle, the electrolysis medium became dark-blue coloured and no deposition product was observed on the electrode surface. Dark blue soluble reaction products were observed, and they diffuse into the bulk solution.

The oxidation processes of sulfonated *N*-alkylaniline take place at higher anodic potential with peak values included in 0.960–1.051 V range, in comparison with *N*-methyl aniline for which it was reported that the CVs show three anodic peaks located at 0.68 V, 0.74 V, and 0.86 V. This can be correlated with the presence of the bulky *N-*propane sulfonic acid group. The sulfonic group (–SO_3_^−^) attached to the nitrogen atom has a strong electron-withdrawing character, and in combination with the steric effects, will increase the oxidizing potential of the aniline monomer [34].

**Figure 6:**
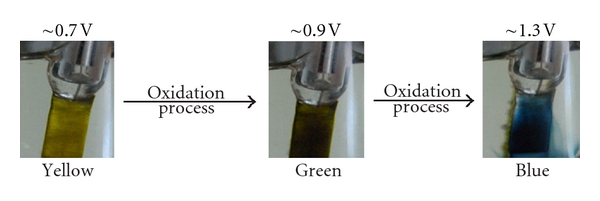
The colour changes by electrooxidation of AnPS in acidic aqueous medium.

During the oxidation process, the AnPS monomer exhibits an electrochromic behaviour, being yellow at low potential value and passing through green and blue to black at higher potentials (up to 1.0 V) (Figure 6).

### 3.2. Electrochemical Deposition of Copolymers Thin Layers

The simple and efficient way to obtain sulfonated self-doped polyanilines, possessing different solubility and enhanced properties, is chemical or electrochemical *co*polymerization reactions. Sulfonated* co*polyanilines can be obtained by choosing the ratio between monomers and appropriate (electro)polymerization conditions [27].

In an attempt to synthesize AnPS, by taking an excess of aniline in reaction with 1, 3-propanesultone, a mixture of AnPS and aniline was obtained that couldnot be separated by ordinary separation techniques. An explication for this can be the coexistence of the AnPS and aniline monomers as an acid-base complex in which the NH_2_ group of aniline is protonated by the sulfonic acid of AnPS. The ratio between these two monomers was calculated from the ^1^H NMR spectra and is 1 : 0.75 AnPs to aniline. By submission of this monomer mixture to electrochemical measurements, the cyclic voltammograms recorded with a Pt plate and ITO-glass as working electrodes in H_2_SO_4_ (1 M) aqueous solution present on the first cycle one anodic peak and two cathodic peaks which correspond to oxidation and reduction processes of the AnPS monomer.

**Figure 7:**
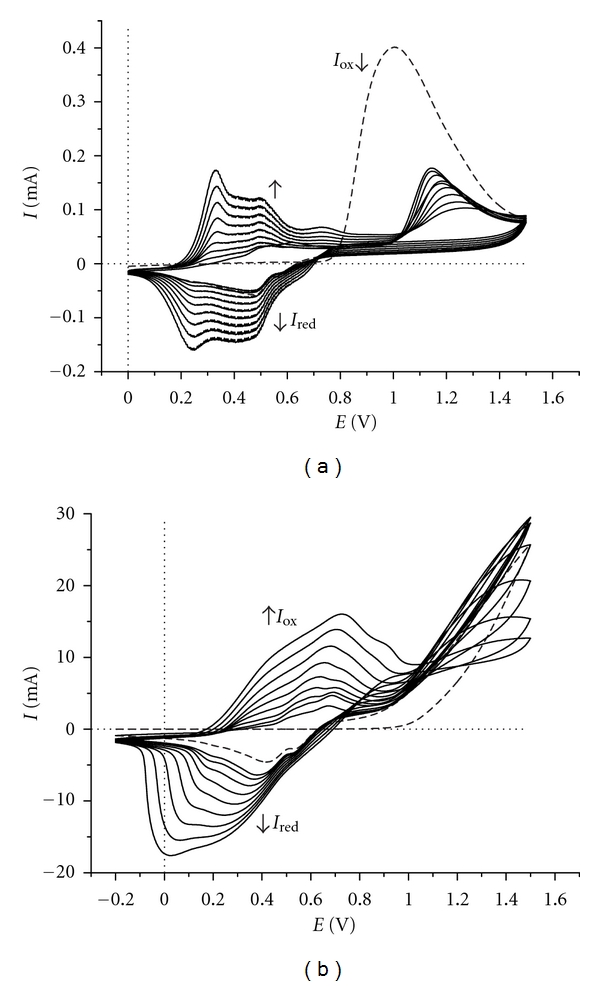
Multicyclic voltammograms (20 cycles) of AnPS-aniline mixture (10^−2 ^M) in H_2_SO_4_ (1 M) aqueous solution at (a) Pt plate (1 × 0.5 cm^2^) and (b) ITO glass (2.5 × 2.5 cm^2^) working electrode; the dotted line represents the first cycle. Scan rate: 50 mV s^−1^.

In case of a mixture containing both AnPS and aniline is expected that the electrooxidation and the polymerization process, respectively, be more complex because, besides the anodic oxidation and chemical coupling of AnPS monomer the redox processes characteristic of aniline must be taken into account. From the cyclic voltammograms recorded at Pt plate electrode (Figure 7(a)) it can be seen that during the first anodic scan a single peak at 1.008 V and two cathodic peaks at 0.581 V and 0.489 V appear. These peaks are characteristic to oxidation and reduction of AnPS monomer and in the presence of a small amount of aniline the cation-radical formed in the first oxidation process represents the promoting intermediate of the electro-*co*-polymerization reaction. By increase the number of the scans the current intensities of redox couples increases and new oxidation and reduction peaks appears. The appearance of new oxidation and reduction peaks at lower values of potentials and the increasing of the current intensities confirm the deposition of a thin layer of polymer on the working electrode surface.

 Overall, the electrochemical growth of the copolymer on the working electrode employing cyclic voltammetry is significantly different in the electrochemical behaviour of the AnPS and aniline individually. Moreover, the appearance of new redox couple 0.330 V/0.250 V in the cyclic voltammograms of the AnPS: aniline mixture marks the formation of a thin layer of copolymer because this does not appear in the cyclic voltammograms of the deposited polyaniline. The cyclic voltammograms recorded at ITO-glass electrode are characterized by oxidation and reduction large peaks and their current intensities increase with the number of scans (Figure 7(b)). In both cases, at the end of the 20th cycles, on working electrode surface a thin layer of blue-green polymer was observed.

**Figure 8:**
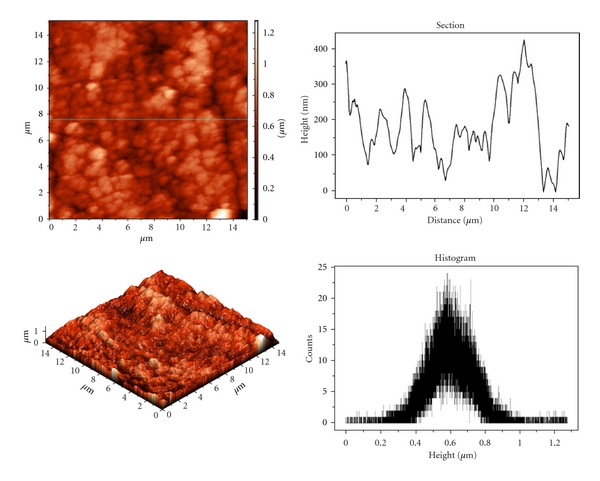
AFM images (2D and 3D) and the section profile of the thin layer of poly(AnPS-*co*-An) deposited on the Pt electrode surface by cyclic voltammetry.

The data regarding the morphology and roughness of the thin layer deposited on Pt electrode was provided by AFM technique. In Figure 8 the thin layer topography (2D and 3D images) and section profile can be seen. It can be observed that the organization of the polymer structure formed by electrochemical polymerization is very compact and “granular”, and granules formed have an uneven distribution of size, and the roughness layer is high. The data provided by the software device confirm the higher roughness of the layer, being characterized by the following parameters: root mean square (Sq) = 128.355 nm and average roughness (Sa) = 100.538 nm.

**Figure 9:**
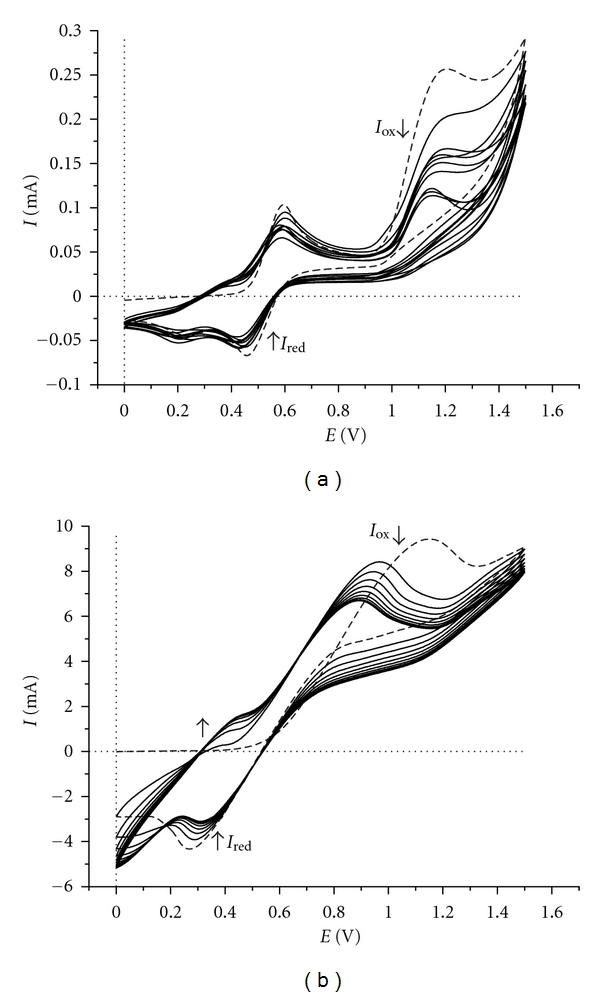
Multicyclic voltammograms (20 cycles) of DAnPS-aniline (1 : 3 ratio mixture) (10^−2 ^M) in DMF-HCl (1 M) aqueous solution at (a) Pt plate (1.0 × 0.5 cm^2^) and (b) ITO glass (2.5 × 2.5 cm^2^) as working electrodes; the dotted line represents the first cycle. Scan rate: 50 mV s^−1^.

This thin green copolymer layer deposited on Pt electrode was not soluble in any of the organic solvents. Trying to obtain soluble polymer a mixture consisting of DAnPS and aniline as comonomers was submitted on cyclic voltammetry measurements, with 1 : 1 and 1 : 3 feed ratios. The cyclic voltammograms recorded at Pt electrode and transparent ITO-glass electrodes in DMF-HCl (1 M) aqueous solution are presented in Figure 9. In the first case, at the echimolar ratio between monomers, no polymer layer was observed on the electrode surface at the end of the 20th cycles. A thin copolymer layer deposited on the electrode surface by cycling the potential between 0.0 V and 1.5 V in DMF-HCl aqueous solution (1 M) was observed when the ratio between DAnPS and aniline was 1 : 3.

The recorded cyclic voltammograms are irreversible. The mechanism of electrochemical polymerization of this mixture is very complex, and thus, it cannot be stated if DAnPS monomer is responsible for the appearance of these peaks. Continuing with the scanning on the same potential range, the redox current intensities fall gradually during the 10 cycles. At the end of the 10th cycles a blue-green thin layer copolymer was observed deposited on the Pt electrode surface.

All attempts to get an adherent copolymer layer at the electrode surface by using the same system but from the aqueous H_2_SO_4_ (1 M) solution failed because, at the end of 10 cycles, the reaction products formed at the electrode are dispersed in solution. In Figure 9(b) are presented the cyclic voltammograms recorded at transparent ITO-glass electrode. Potential-current intensity curves are characterized by anodic and cathodic peaks; both are broad, with the maximum shifted slightly in positive direction for anodic peak and negative values for cathodic peak. The recorded cyclic voltammograms are irreversible. Anode and cathode currents intensities decrease slightly with increasing of the number of scans.

**Figure 10:**
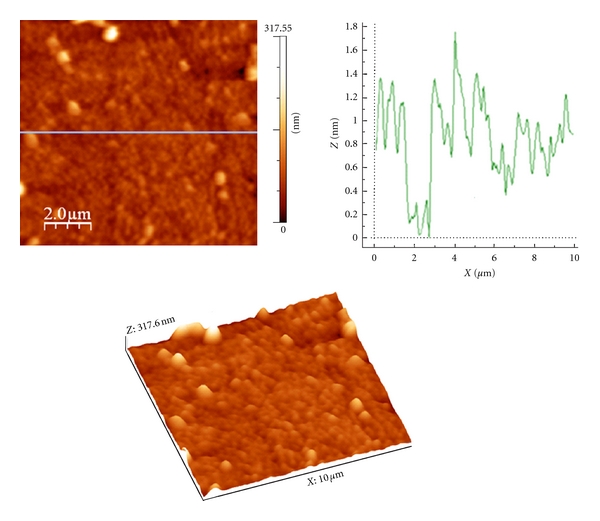
AFM images (2D and 3D) and the section profile of the thin layer of poly(AnPS-*co*-An) deposited on the Pt electrode surface by cyclic voltammetry.

The poly(DAnPS-*co*-An) deposited on ITO-glass electrode surface by electrochemical polymerization has a dense and compact structure with granular morphology, whose size has a uniform distribution (Figure 10). Data provided by the AFM software device shows that, compared with the precedent case (poly(AnPS-co-An)), the thin layer of poly(DAnPS-co-An) has a smaller roughness which is characterized by the following parameters: root mean square (Sq) = 22.45 nm and average roughness (Sa) = 14.41 nm.

**Figure 11:**
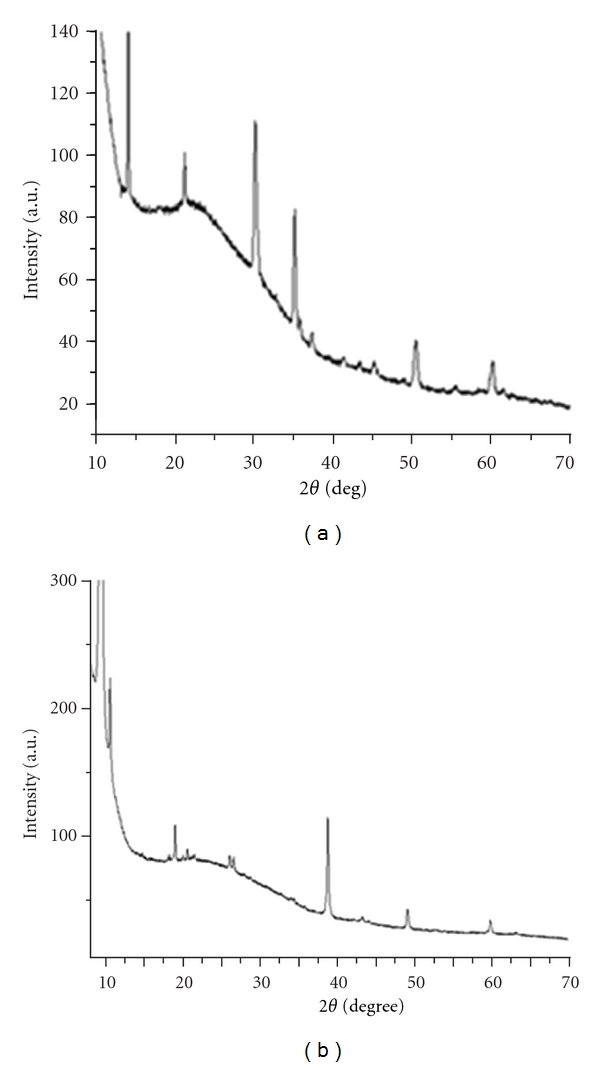
XRD spectra of poly(AnPS-*co*-An) (a) and poly(DAnPS-*co*-An) (b).

Analyzing the thin layers by X-ray technique, results that both copolymers (poly(AnPS-*co*-An) and poly(DAnPS-*co*-An)) obtained by deposition on glass-ITO electrode by electrochemical polymerization present a neat structure with crystalline regions (Figure 11).

The development of the highly ordered structures for self-doped poly(aniline-N alkanesulfonate)s was evidenced by Kim et al. [28]. The copolymers formed on working electrodes, by electrochemical synthesis, are characterized by a highly orientational and positional order associated with ionic interactions between alkylsulfonates groups and polyaniline main chain. Our group synthesized homopolymers and copolymers based on *N-*(3-sulfopropyl) aniline acid by chemical oxidative polymerization, and the XRD analysis revealed that the homopolymers and even the copolymers have a highly ordered structure indicating a high degree of crystallinity [27].

## 4. Conclusions

In summary, the cyclic voltammetry was used to study the electrochemical behaviour of two *N*-substituted aniline derivatives containing sulfonic acid group attached at the nitrogen atoms through a spacer consisting of a three methylene units. The cyclic voltammetric measurements were carried out in three electrolytic aqueous solutions such as 1 M H_2_SO_4_, 1 M HCl, and 1 M HClO_4_, employing two working electrodes such as Pt (disk-shaped and rectangular plate) and transparent ITO-glass electrode. Due to the self-doping effect of the sulfonic groups, AnPS and DAnPS (partially) have good electroactivity in neat water solution. Experimental results showed that both aniline derivatives are electroactive species exhibiting redox peaks in the −0.2 V and 1.6 V potential ranges, and the shape of CVs recorded, and the redox process of aniline derivatives, respectively, are influenced by the anions nature. During the anodic oxidation processes noticeable colour changes at the electrode take place while soluble reaction products are formed and diffuse into the bulk solution. Due to the high solubility of oligomers formed during the oxidation processes, no homopolymer deposited on the electrode was observed. By adding small amount of aniline monomer into electrolytic system, thin, compact, and crystalline films of copolymers were deposited on the working electrode surface.

## Figures and Tables

**Table 1:** Cyclic voltammetry data of DAnPS and AnPS recorded at Pt electrode in different electrolyte media.

*E*_*p*_ (V)/*I*_*p*_ (*μ*A)	DAnPS (1.0 mM)					AnPS (1.0 mM)		
	H_2_O (dispersion)	DMF-LiClO_4_	DM-H_2_SO_4_ (1 M)	DM-HClO_4_ (1 M)	DMF-HCl (1 M)	H_2_SO_4_ (1 M)	HClO_4_ (1 M)	HCl (1 M)
*E*_ox_^onest^/*I*_ox_^onest^	0.118/0.30	0.409/1.6	0.445/2.29	0.437/2.20	0.432/2.59	0.805/3.17	0.773/3.51	0.777/0.43 × 10^3^
*E*_ox_^1^/*I*_ox_^1^	0.569/9.01	0.525/33.5	0.552/29.33	0.544/30.62	0.529/21.68	0.983/26.78	0.960/49.06	1.051/12.59 × 10^3^
*E*_ox_^2^/*I*_ox_^2^	—	1.027/22.8	1.075/19.96	1.064/23.62	1.474/−4.88	—	—	1.363/12.36 × 10^3^
*E*_red_^onest^/*I*_red_^onest^	0.424/1.42	0.560/1.3	0.593/−0.61	0.583/2.06	0.925/18.29	0.580/−2.66	0.594/−3.92	0.552/−0.27 × 10^3^
*E*_red_^1^/*I*_red_^1^	0.145/−3.22	0.435/−30.0	0.454/−25.28	0.455/−27.13	0.937/−18.22	0.399/−13.60	0.522/−11.68	0.390/−1.76 × 10^3^
*E*_red_^2^/*I*_red_^2^	−0.091/−5.14	—	after 3rd cycle 0.207	—	0.431/−20.87	—	0.426/−22.57	—
*I*_ox_/*I*_red_	1.75	1.11	1.16	1.17	0.99	1.97	4.20	—
